# Love your wet grass! Dry season grazing reserves show highest grass regrowth in communal semi-arid rangelands of Tanzania

**DOI:** 10.1371/journal.pone.0313818

**Published:** 2024-11-18

**Authors:** Sabine A. Baumgartner, Stuart W. Smith, Gundula S. Bartzke, Oloshiro Laizar, Jane F. Ploechl, Lena M. Michler, Elizabeth M. Naro, Anna C. Treydte

**Affiliations:** 1 Department of Ecology of Tropical Agricultural Systems (490f), Institute of Agricultural Sciences in the Tropics (Hans-Ruthenberg-Institute), University of Hohenheim, Stuttgart, Germany; 2 Department of Physical Geography, Stockholm University, Stockholm, Sweden; 3 Ecological Sciences, The James Hutton Institute, Craigiebuckler, Aberdeen, United Kingdom; 4 Biostatistics Unit, Institute of Crop Science, University of Hohenheim, Stuttgart, Germany; 5 Grazing Committee, Village of Loibor Siret, Tanzania; 6 Istituto Oikos, Arusha, Tanzania; 7 African People & Wildlife Fund, Bernardsville, NJ, United States of America; U.S. Geological Survey, UNITED STATES OF AMERICA

## Abstract

Land management by seasonal migratory herding, traditionally implemented by pastoralists, has allowed semi-arid rangeland ecosystems to remain productive and resilient to highly erratic, seasonal rainfall patterns. Changing pastoralist practices and rainfall patterns due to climate change have the potential to negatively influence the resilience of rangelands. To test the impact of different communal rangeland practices and increasing frequency of disturbance on the rangelands’ vegetation, we established a clipping experiment in three rangeland management types: rainy season rangeland, dry season rangeland and seasonal exclosures, in the Maasai Steppe, northern Tanzania. Across these rangeland management types, we tested two clipping frequencies monthly vs. once per growing period and either fenced or open to grazing (herbivory) over two consecutive growing periods. We found that rainy season rangeland exhibited lowest regrowth rates and highest proportion of bare ground across herbivory, clipping frequency and growing period. When rainfall was low, seasonal exclosures showed lowest proportion of bare ground across herbivory and clipping frequency and plots that were seasonally clipped and excluded herbivores generated significantly more grass biomass, higher grass regrowth rates and lowest proportion of bare ground compared to all other treatments. Excluding herbivores had little impact on the vegetation biomass and cover but had a positive impact on grass regrowth rates when rainfall was high. Seasonal clipping allowed for lower proportion of bare ground, and in most cases higher regrowth rates and higher biomass compared to monthly clipping. We conclude that traditional seasonal migratory herding is a relevant concept to sustain rangeland productivity and resilience under increasing intensity of disturbance if grazing pressure on rainy season rangelands is adapted to rainfall conditions and sufficient resting time after heavy defoliation is granted. Dry season grazing (including rainy season resting) proved to be the most sustainable concept to maintain regrowth and control erosion.

## Introduction

### General introduction

Arid and semi-arid rangelands cover more than two-thirds of Africa’s land surface, where a large proportion of the pastoralists and agropastoralists rely on rangelands for their livelihood. The current rapid rangeland degradation, due to unsustainable use by people and livestock and an increased climate variability, affects more than half of the continent’s population [1, 2]. Adaptive grazing management of rangelands is an integral part of maintaining grazing resources for livestock [3]. One such adaptive grazing management is transhumance, or seasonal migratory herding in response to climatic and forage conditions, which is a traditional pastoralists practice in semi-arid rangelands worldwide (e.g. [4, 5]). Transhumance has been a sustainable, climate-smart grazing management strategy applied for centuries [5]. However, political and environmental land-use policies have resulted in severe land fragmentation, encouraged sedentarisation and increased the pressure on the remaining rangelands of East Africa over the last few decades [6, 7]. Increasing fragmentation of the landscape will likely further restrain the mobility of pastoralists and make them and the rangelands more susceptible to changing and more severe rainfall patterns in the future [8, 9]. These processes make it imperative to quantify the relevance of traditional management systems in the combat against the deterioration of valuable grazing resources and how resilient the rangelands are towards increasing disturbance.

### How suitable is traditional grazing management nowadays?

Transhumance is a diverse and highly adaptable strategy, in terms of grazing length and regarding climatic conditions, to sustain the regrowth potential of rangelands. Transhumant pastoralists move between predetermined seasonal grazing sites depending on the rainfall, forage availability and quality as well as nutrient requirements of the livestock, thereby adapt to unfavorable climatic conditions [5, 7, 10]. The pastoralists follow yearly movement patterns and use pre-defined dry season (open for livestock grazing during the dry season) and rainy season (open for livestock grazing during the rainy season) rangelands, which enables these rangeland areas to recover during the following dry/ rainy season, respectively [3]. Dry season only grazing promotes undisturbed regrowth of the grass layer during the following rainy season and helps to maintain the rangeland quality in terms of forage composition and productivity. In contrast, vegetation of rangelands, grazed during the rainy season and rested during the dry season, when water is limited, might not be able to build up sufficient reserve biomass and therefore inherit lower regrowth capacity after subsequent defoliation [11–13]. Temporal removal of livestock from seasonal exclosures, serving as forage reservoir for young and weak livestock during the dry season, is another widespread management tool in Sub-Saharan Africa [14–16]. These temporary exclosures can help to increase standing plant biomass and vegetation cover and to conserve herbaceous plant species diversity [14, 15, 17]. While semi-arid rangelands overall seem to be able to recover from disturbance, which reduces degradation in the long run, increasing frequency of disturbance might diminish this capacity [18–20]. In addition, pastoralists’ movement on a large scale it is increasingly restricted by landscape fragmentation and vegetation recovery strongly depends environmental conditions, such as rainfall, during the recovery phase [8, 20]. This leads to the questions of whether the approach of small-scale subdivision of grazing land into dry season and rainy season rangeland and seasonal exclosures, is a suitable concept for maintaining rangeland regrowth capacity under increasing frequency of disturbance, and how climatic conditions, during livestock exclusion affects the rangeland vegetation.

### How do we manage seasonally used rangelands?

Herbivores affect rangeland vegetation by trampling, defoliation and dung deposition [21]. Continuous heavy grazing by livestock can reduce rangeland productivity, grass cover and regrowth [22, 23]. The regrowth capacity of the vegetation after disturbance is a crucial factor that helps to quantify the resilience of rangeland productivity, where declining rangeland productivity and declining vegetation cover are considered indicators for soil and rangeland degradation [23–27]. On the other hand, exclusion of herbivores and underutilization of rangelands may hamper the potential of herbaceous vegetation to regrow after intense disturbance, which is not desirable either [23]. Herbivory can stimulate vegetation growth that compensates for the removed biomass, if intense grazing is followed by a recovery phase [28]. Long-term exclusion of herbivory has been studied intensively, e.g. [25, 29], but to manage communal rangelands on a daily basis, it is crucial to know the required recovery time after heavy disturbance and how herbivores and herbivore exclusion influence the recovery potential. Studies on communally managed semi-arid rangelands usually focus on the rangeland condition by observational studies. Experimental studies, actively modifying the vegetation in the field to measure a diverse array of traditional grazing strategies and their regrowth capacity under different herbivore usage are rare.

We aim to determine the potential of traditional grazing management to maintain plant regrowth in an intensely grazed ecosystem, the Simanjiro Plains in Tanzania. The regrowth capacity of the vegetation after disturbance is often tested using clipping experiments [18, 19]. We measured plant biomass and cover, regrowth capacity as well as proportion of bare ground as indicators for the rangeland’s resilience and recovery potential. We applied a clipping experiment across three rangeland areas traditionally used by the Maasai pastoralists: Seasonal exclosures (used during dry season for young and weak livestock, blocked during the rainy season), dry season rangeland (communal rangeland used only during the dry season) and rainy season rangeland (communal rangeland used only during the rainy season) to verify the following assumptions:

1. Seasonal exclosures and dry season rangeland will show higher resilience of herbaceous vegetation, measured as herbaceous vegetation regrowth and proportion of bare ground, compared to rainy season rangeland, as plants have the longest and wettest periods to recover in the former.
2. Herbaceous plants are more resilient when grazed compared to excluding herbivores.
3. Monthly clipping, resembling the current near-to permanent disturbance of communal areas will diminish the capacity of herbaceous vegetation to recover compared to seasonal clipping.

By monitoring the regrowth capacity and ground cover under community-led grazing management, this study will help inform other pastoral communities and researchers wishing to evaluate and implement grazing management programmes. Our study will also support site- based management decisions and help to reduce the risk of rangeland degradation.

## Methodology

Our field study was carried out with permission of the Tanzania Wildlife Research Institute (TAWIRI) and the Tanzania Commission for Science and Technology (COSTECH; permit no.: 2018-450- NA-208- 225 and 2020-013- NA-2018-225) and with the approval of the Simanjiro District Council in Orkesumet and the local authorities of the Loibor Siret village. No endangered or protected species were sampled during this study. The study area was not protected.

### Study site

**Fig 1:**
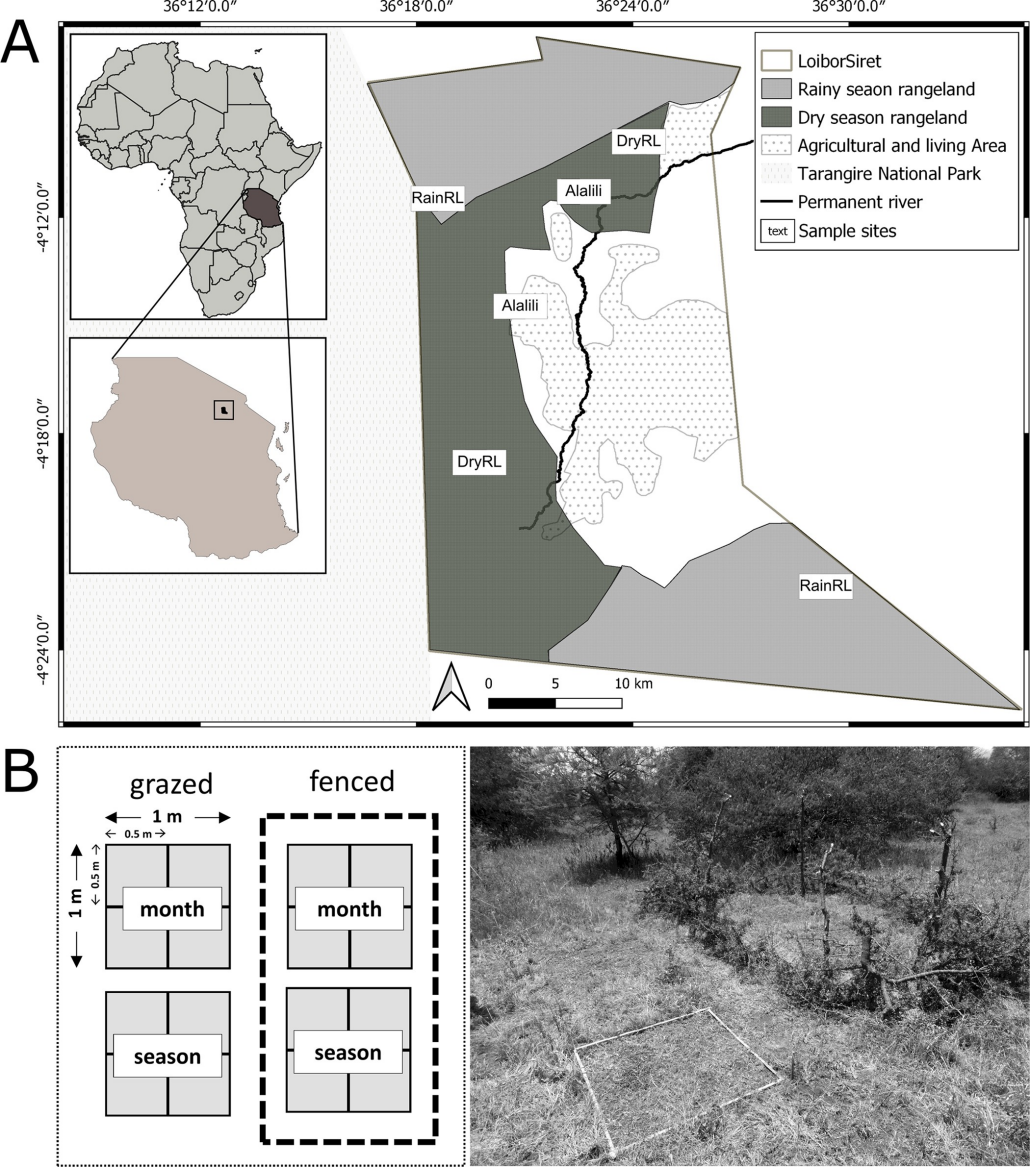
Location of sample sites in the community of Loibor Siret, Tanzania. A) Locations of sample sites in seasonal exclosures (Alalili), dry season rangeland (DryRL) and rainy season rangeland (RainRL) of the community of Loibor Siret, Tanzania. This simplified map was created through participatory mapping including information by the community grazing committee and our partner NGO “African People & Wildlife”. B) Experimental blocks established at our sample sites with quadrats, containing plots, protected by a fence (fenced) or quadrats, containing plots, open for grazing (grazed). Half of the quadrats, containing plots were clipped every month (month), the other half were clipped after one growing period (season). The map was created using the free and open source QGIS version 3.10.4.

The study sites were located in the Simanjiro district of the Maasai Steppe, northern Tanzania (Fig 1). The Simanjiro region is characterized by undulating plains of semi-arid savanna landscapes receiving bi-modal average annual rainfall of 400–800 mm with high interannual variation comprising short rains from November till January and long rains from February/March till May [30]. The soils on hill slopes and crests mainly consist of dark red sandy clay loams, whereas flood plains are characterized by clay and organic rich soil, locally known as black cotton soils [31]. The main vegetation types of the Simanjiro Plains include grassland areas dominated by *Digitaria macroblephara*—*Panicum coloratum* grassland intermixed between *Vachellia tortillis*—*Commiphora schimperi* woodland and *Vachellia stuhlmannii*—*Vachellia drepanolobium* bushland. For a detailed description of the vegetation in the Simanjiro Plains see [32].

Livestock pasture is the dominant land-use, covering grass-, wood- and bushlands and 10% of the area is used for agriculture, the latter likely to expand in the near future [33]. Livestock herds consist of 75% cattle, i.e., Maasai zebu (breed within the zebu cattle *Bos indicus*, [34]), followed by sheep (*Ovis aries*, breed Red Maasai, Blackhead Somali) and goat (*Capra hircus*, breed Small East African, Maasai) [33]. Livestock numbers in the Simanjiro Plains have increased steadily at a high rate [35]. The area experiences frequent visits by wildlife and plays an important role in wildlife conservation as corridor for the seasonal migration from the adjacent Tarangire National Park [6]. Wild herbivores in the Simanjiro Plains mainly consists of Cape buffalo (*Syncerus cafer*), zebra (*Equus burchellii)*, giraffes (*Giraffa camelopardalis*), wildebeest (*Connochaetes taurinus*), impala (*Aepyceros melampus*), Grant’s gazelle (*Gazella granti*) and Thomson’s gazelle (*Gazella rufifrons*) [35].

In 2012, the community of Loibor Siret introduced a land-use management plan and divided the communal rangeland into two rangeland areas based on the traditional Maasai land- use system (e.g., [36]: (1) rainy season rangeland (from now on referred to as ‘RainRL’) grazed for up to seven months between December to July, (2) and dry season rangeland (from here on referred to as ‘DryRL’) grazed for up to five months between July and December [37, 38]. Additionally, the Maasai temporarily deny access of livestock to (3) seasonal exclosures (from now on referred to as ‘Alalili’) that only open during the dry season and are restricted to young and weak livestock only (Fig 1) [39].

At the beginning of 2019, the overall livestock stocking rate on the openly accessible community rangeland (including DryRL and RainRL) of Loibor Siret was roughly 1 tropical livestock uni (TLU)/ha for cattle and 0.1 TLU/ha for sheep and goats (shoats) [37]. Grazing pressure and stocking rates usually vary, depending on the distance to water resources [40], and distance from living areas [4]. In the studied Alalili, stocking rates of cattle and shoats were roughly 0.5–1.2 TLU/ha and 0.15–0.6 TLU/ha, respectively (numbers derived from the head of the homestead (boma) and the Grazing Committee of the community of Loibor Siret [37]).

### Experimental design and data collection

In 2019, we established a clipping experiment within each rangeland area (Alalili, DryRL and RainRL) and selected two replica sites per rangeland area (see Fig 1A). Sites were selected to be as similar as possible in terms of environmental conditions, such as soil type, gradient and position on the slope. Grazing land on vertisols (black cotton soil) were excluded from the study. At each site, we established two blocks that included a fully- factorial design of all treatments. Each treatment was applied to four 0.5 x 0.5 m plots located within a 1 x 1m quadrat (see Fig 1B). The location of the blocks was selected randomly and under the premise that they were not influenced by a tree canopy or dominated by herbs or shrubs. Quadrats within the block were either fenced with metal wires and protected by thorny branches, excluding herbivores larger than 5 kg (from here on referred to as ‘fenced’) or open to herbivore grazing (from here on referred to as ‘grazed’). Additionally, we applied two different clipping frequencies: either every month (from here on referred to as ‘month’) or only once after three months, which covers the duration of one growing season (from here on referred to as ‘season’). Thus, our treatments consisted of four combinations: 1.) excluding herbivory and monthly clipping (fenced, month); 2.) excluding herbivory and seasonal clipping (fenced, season); 3.) open for grazing and monthly clipping (grazed, month), 4.) open for grazing and seasonal clipping (grazed, season). In total, we monitored vegetation parameter of 192 plots. The clipping experiment was repeated for two consecutive growing periods: the first growing period between March and May 2019 (three months) and the second growing period between November 2019 and January 2020 (three months). During growing period 1, rainfall was rather low totaling 234 mm which is about half of the long-term average for those particular months. During growing period 2 rainfall was exceptionally high, totaling roughly 510 mm, which is about 88% higher than the long term average for those months (rainfall data 2019/2020 calculated in Google Earth Engine using the Climate Hazards Group InfraRed Precipitation with Station data (CHIRPS) for the adjacent Tarangire National Park [30, 41, 42]).

At the beginning of the experiment (original biomass) and at the beginning of the second growing period, the herbaceous vegetation was clipped to 2.5 cm above the ground surface [43]. Clipped biomass was separated into plant life-form groups: (1) grasses and sedges, according to [44] (hereafter ‘grass’), and (2) herbs and shrubs as specified in [45] (hereafter ‘forbs’). Samples were sundried for seven days, and then oven-dried at 60°C for 24 hrs until constant weight and finally weighed (± 0.01 g). Monthly clipped biomass was summed for the entire growing period (cumulative biomass) and compared to seasonally clipped biomass. We calculated grass or forb regrowth capacity by subtracting the regrown cumulative biomass from the original biomass, the latter being obtained by the initial cut of the vegetation in our plots in March 2019, before treatment application (*Regrowth capacity = Regrown biomass–original biomass*; positive regrowth relates to more biomass than originally found; negative regrowth relates to less biomass than originally found; (see [23]). Regrowth capacity was used as an indicator for the resilience of rangeland productivity. Prior to clipping, grass and forb cover, and the proportion of bare ground was estimated to the nearest 5% in each plot [46]. We are aware that our clipping experiments did not consider all nuanced effects of herbivory, such as trampling, dung/ urine deposition or selective grazing, but focused on the impact of biomass removal. Therefore, the results might not reflect the full impact of intense herbivory. However, this study will reveal crucial insight into the rangelands’ regrowth and recovery potential after heavy disturbance.

### Statistical analyses

We applied seven linear and generalized linear mixed-effects mixed models per growing period to analyze the effect of the fixed factors ‘rangeland area’ (Alalili vs. DryRL vs. RainRL), ‘herbivory’ (fenced vs. grazed), and ‘clipping frequency’ (monthly vs. seasonally clipped) on the dependent variables ‘grass biomass’ and ‘forb biomass’, ‘grass regrowth’ and ‘forb regrowth’, ‘grass cover’, ‘forb cover’ and the ‘proportion of bare ground’ respectively. Fixed factors were considered for the model based on our hypotheses that rangeland area, herbivory and clipping frequency exert strong influence on biomass regrowth and cover [12, 14, 23, 47]. We analyzed two way- interaction of the fixed factors, as we assumed that the three rangeland areas have distinct coping potentials towards herbivory and that clipping frequency affects the vegetation differently, depending on herbivore presence. We applied the full model including all fixed factors and their two-way interaction, since we considered all of them important to answer our research questions. To account for repeated measurements from the same site, block and quadrat, we nested ‘quadrat’ in ‘block’ in ‘site’, and included them in the model as nested random factors. We used the above described full model structure for all models. Biomass and regrowth were analyzed using linear mixed-effects models with a Gaussian distribution. Vegetation cover data (‘grass cover’, ‘forb cover’, ‘proportion of bare ground’) were analyzed using generalized linear mixed-effects models with a beta- distribution set between 0 and 1, for 0 and 100% plant cover, respectively, as generalized linear mixed-effects models can handle non-Gaussian response variables [48]. Clipping was conducted on the same plots throughout both growing periods. Therefore, we analyzed the results of the two growing periods separately [49]. The cover data used for analyses were measured before the final cut at the end of each growing period. We conducted analyses of deviance using chi-squared tests for cover data and t-tests/ AONVA for the other metric variables. If the p-value for interactions between the fixed factors was less than 0.05, we conducted additional post-hoc analyses using Tukey’s adjustment [50]. Post-hoc analyses were also applied when fixed factors had more than two levels. We checked the residuals of our data for homogeneity of variance and normal distribution, except for the beta-distribution models. Where residuals did not fulfil the criteria of normal distribution we transformed the data using a Cox-Box- transformation (0.04 ≤ λ ≤ 0.42; [51]). The standard error of Cox-Box- transformed variables were calculated applying the delta method. Results are given as mean ± standard error. Statistical analyses were performed using R statistical software 3.6.3 with the packages ‘geoR’, ‘lme4’, ‘lmerTest’, ‘emmeans’ ‘glmmTMB’ and ‘car’ and ‘carData’ in R studio [52–60]. Significance level was set at α = 0.05. Graphs were created using QtiPlot version 5.12.8 and processed using Inkscape version 0.92.4.

## Results

### Rainy season rangeland shows lowest plant cover and regrowth capacity (H1)

**Fig 2:**
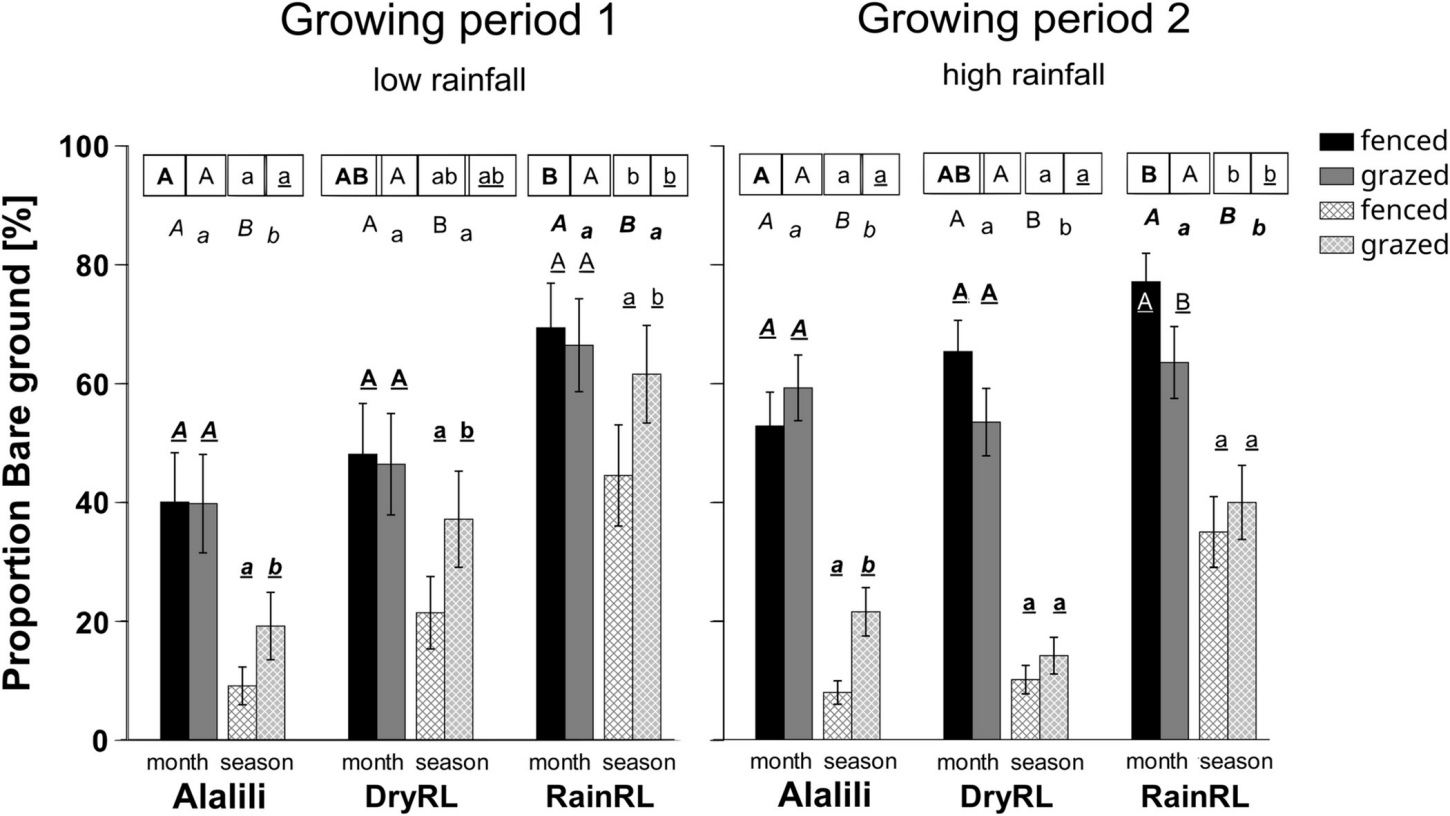
Proportion of bare ground in experimental plots. Mean proportion bare ground at the end of the growing period [%] in experimental plots in the rangelands of the community of Loibor Siret, Tanzania. Experimental plots were monitored across three different rangeland areas: Seasonal exclosures (Alalili), dry season rangeland (DryRL) and rainy season rangeland (RainRL). Plots were clipped each month (‘month’, black and grey bars) or once after the growing period (‘season’ crosshatched bars). Half of the quadrats, containing plots, were fenced to exclude herbivores (‘fenced’, black bars and white bars with grey crosshatches), the other half were open for grazing (‘grazed’, grey bars and grey bars with white crosshatches). The graphs show the results of two growing periods: Growing period 1 (March- June 2019) and Growing period 2 (November 2019 –January 2020). Significant differences (p<0.05) are indicated by different letters of same style. Letters in squared boxes show differences between rangeland areas. Letters without boxes and not underscored show differences between clipping frequencies month and season. Underscored letters show differences between fenced and grazed plots. Vertical bars represent ± 1 s.e.

**Fig 3:**
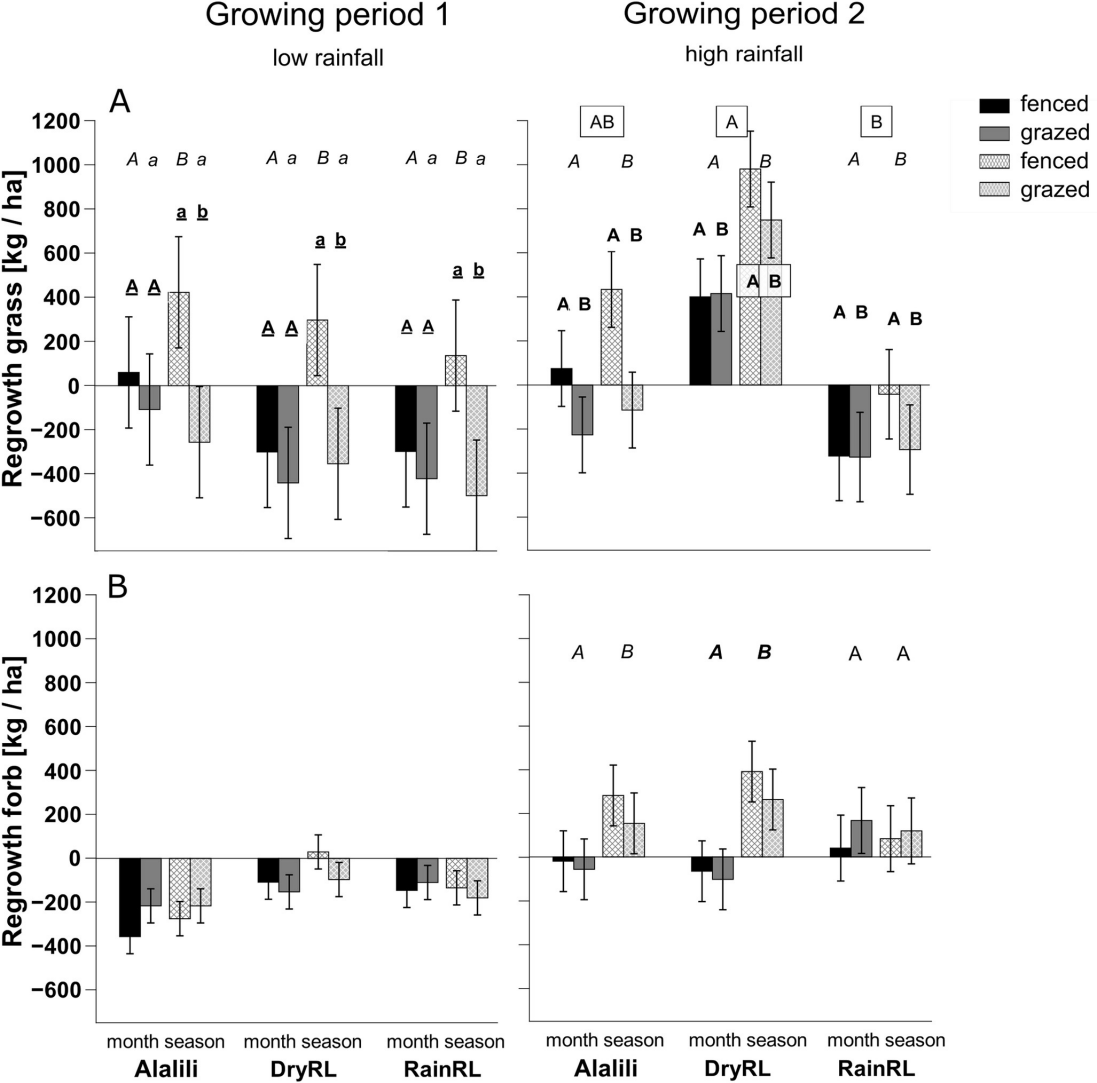
Regrowth of grass and forbs in experimental plots. **(A)** Cumulative grass regrowth and **(B)** cumulative forb regrowth per season [kg/ ha] (positive values relate to more biomass regrowth than original biomass, and negative values relate to less regrowth than original biomass) for experimental plots in the rangelands of the community of Loibor Siret, Tanzania. Experimental plots were set up in the three different rangeland areas: Seasonal exclosures (Alalili), dry season rangeland (DryRL) and rainy season rangeland (RainRL). Plots were clipped each month (‘month’, black and grey bars) or once after the growing period (‘season’ crosshatched bars). Half of the quadrats, containing plots, were fenced to exclude herbivores (‘fenced’, black bars and white bars with grey crosshatches), the other half were open for grazing (‘grazed’, grey bars and grey bars with white crosshatches). The graphs show the results of two growing periods: Growing period 1 (March- June 2019) and Growing period 2 (November 2019 –January 2020). Significant differences (p<0.05) are indicated by different letters of same style. Letters in squared boxes show differences between rangeland areas. Letters without boxes and not underscored show differences between clipping frequencies month and season. Underscored letters show differences between fenced and grazed plots. Vertical bars represent ± 1 s.e..

Rainy season rangeland showed highest proportion of bare ground across herbivory, clipping frequencies and the growing periods during both growing periods (Fig 2 and Table 1; additional information on the full models, see S1 Table). During growing period 2, grass regrowth was lowest and, in most cases, negative in RainRL across herbivory and clipping frequency (Fig 3 and Table 1). At the same time, forb regrowth in RainRL was positive throughout all treatments (Fig 3). Alalili showed lowest proportion of bare ground during growing period 1 (Fig 2 and Table 1). The remaining measured variables, such as grass and forb biomass and grass and forb cover did not differ significantly across the rangeland areas during both growing periods (Table 2).

**Table 1:** Factors considered for modelling proportion of bare ground and grass and forb regrowth.

Response variable	GP	factors	F / χ2	*p*	
Proportion Bare ground	GP1	clippingFrequency	62.61	<0.001	***
		herbivory	0.002	0.96	
		rangelandArea	6.81	0.03	*
		clippingFrequency * herbivory	13.95	<0.001	***
		clippingFrequency * rangelandArea	9.11	0.01	*
		herbivory * rangelandArea	0.20	0.90	
	GP2	clippingFrequency	69.68	<0.001	***
		herbivory	0.83	0.36	
		rangelandArea	10.65	0.005	**
		clippingFrequency * herbivory	8.14	0.004	**
		clippingFrequency * rangelandArea	6.70	0.04	*
		herbivory * rangelandArea	7.06	0.03	*
Grass regrowth	GP1	clippingFrequency	5.18	0.03	*
		herbivory	18.84	<0.001	***
		rangelandArea	0.51	0.65	
		clippingFrequency * herbivory	7.72	0.009	**
		clippingFrequency * rangelandArea	0.57	0.57	
		herbivory * rangelandArea	0.02	0.98	
	GP2	clippingFrequency	8.34	0.008	**
		herbivory	5.04	0.03	*
		rangelandArea	12.87	0.003	**
		clippingFrequency * herbivory	1.61	0.22	
		clippingFrequency * rangelandArea	0.85	0.44	
		herbivory * rangelandArea	1.16	0.33	
Forb regrowth	GP1	clippingFrequency	1.30	0.26	
		herbivory	0.01	0.92	
		rangelandArea	2.05	0.28	
		clippingFrequency * herbivory	1.66	0.21	
		clippingFrequency * rangelandArea	1.32	0.28	
		herbivory * rangelandArea	2.83	0.08	
	GP2	clippingFrequency	12.24	0.002	**
		herbivory	0.20	0.66	
		rangelandArea	0.02	0.98	
		clippingFrequency * herbivory	0.52	0.48	
		clippingFrequency * rangelandArea	3.40	0.049	*
		herbivory * rangelandArea	0.67	0.52	

Factors considered for modelling the proportion of bare ground and grass and forb regrowth during two consecutive growing periods (GP). The main factors are ‘clipping frequency’ (month vs. season), ‘herbivory’ (fenced vs. grazed) and ‘rangeland area’ (Alalili vs. DryRL vs. RainRL), as well as the interaction (denoted by ‘*’) between them. Significance codes: <0.001 ‘***’, <0.01 ‘**’ <0.05 ‘*’.

**Table 2:** Factors considered for modelling grass and forb biomass and cover.

Response variable	GP	factors	F / χ2	*p*	
Grass biomass	GP1	clippingFrequency	3.14	0.09	
		herbivory	25.46	< 0.001	***
		rangelandArea	0.54	0.63	
		clippingFrequency * herbivory	9.55	0.004	**
		clippingFrequency * rangelandArea	1.41	0.26	
		herbivory * rangelandArea	0.26	0.77	
	GP2	clippingFrequency	8.61	0.007	**
		herbivory	1.75	0.20	
		rangelandArea	1.22	0.41	
		clippingFrequency * herbivory	1.52	0.23	
		clippingFrequency * rangelandArea	0.65	0.53	
		herbivory * rangelandArea	1.91	0.17	
Forb biomass	GP1	clippingFrequency	0.21	0.65	
		herbivory	2.48	0.13	
		rangelandArea	0.28	0.77	
		clippingFrequency * herbivory	0.72	0.40	
		clippingFrequency * rangelandArea	0.31	0.74	
		herbivory * rangelandArea	0.71	0.50	
	GP2	clippingFrequency	17.39	<0.001	***
		herbivory	2.24	0.15	
		rangelandArea	0.23	0.81	
		clippingFrequency * herbivory	0.0005	0.98	
		clippingFrequency * rangelandArea	3.02	0.07	
		herbivory * rangelandArea	2.04	0.15	
Grass cover	GP1	clippingFrequency	25.41	< 0.001	
		herbivory	0.13	0.71	
		rangelandArea	3.83	0.14	
		clippingFrequency * herbivory	4.82	0.03	*
		clippingFrequency * rangelandArea	3.00	0.22	
		herbivory * rangelandArea	0.06	0.97	
	GP2	clippingFrequency	24.92	< 0.001	***
		herbivory	1.53	0.22	
		rangelandArea	3.29	0.19	
		clippingFrequency * herbivory	2.07	0.15	
		clippingFrequency * rangelandArea	0.18	0.91	
		herbivory * rangelandArea	0.20	0.91	
Forb cover	GP1	clippingFrequency	2.29	0.13	
		herbivory	1.57	0.21	
		rangelandArea	1.30	0.52	
		clippingFrequency * herbivory	1.47	0.22	
		clippingFrequency * rangelandArea	2.82	0.24	
		herbivory * rangelandArea	0.08	0.96	
	GP2	clippingFrequency	33.70	< 0.001	***
		herbivory	3.32	0.07	
		rangelandArea	1.95	0.38	
		clippingFrequency * herbivory	0.006	0.94	
		clippingFrequency * rangelandArea	1.534	0.46	
		herbivory * rangelandArea	5.69	0.06	

Factors considered for modelling grass and forb biomass and grass and forb cover during two consecutive growing periods (GP). Main factors are ‘clipping frequency’ (month vs. season), ‘herbivory’ (fenced vs. grazed) and ‘rangeland area’ (Alalili vs. DryRL vs. RainRL), as well as the interaction (denoted by ‘*’) between them. Significance codes: <0.001 ‘***’, <0.01 ‘**’ <0.05 ‘*’.

### Herbivory affects vegetation parameters strongly when rainfall is low (H2)

**Fig 4:**
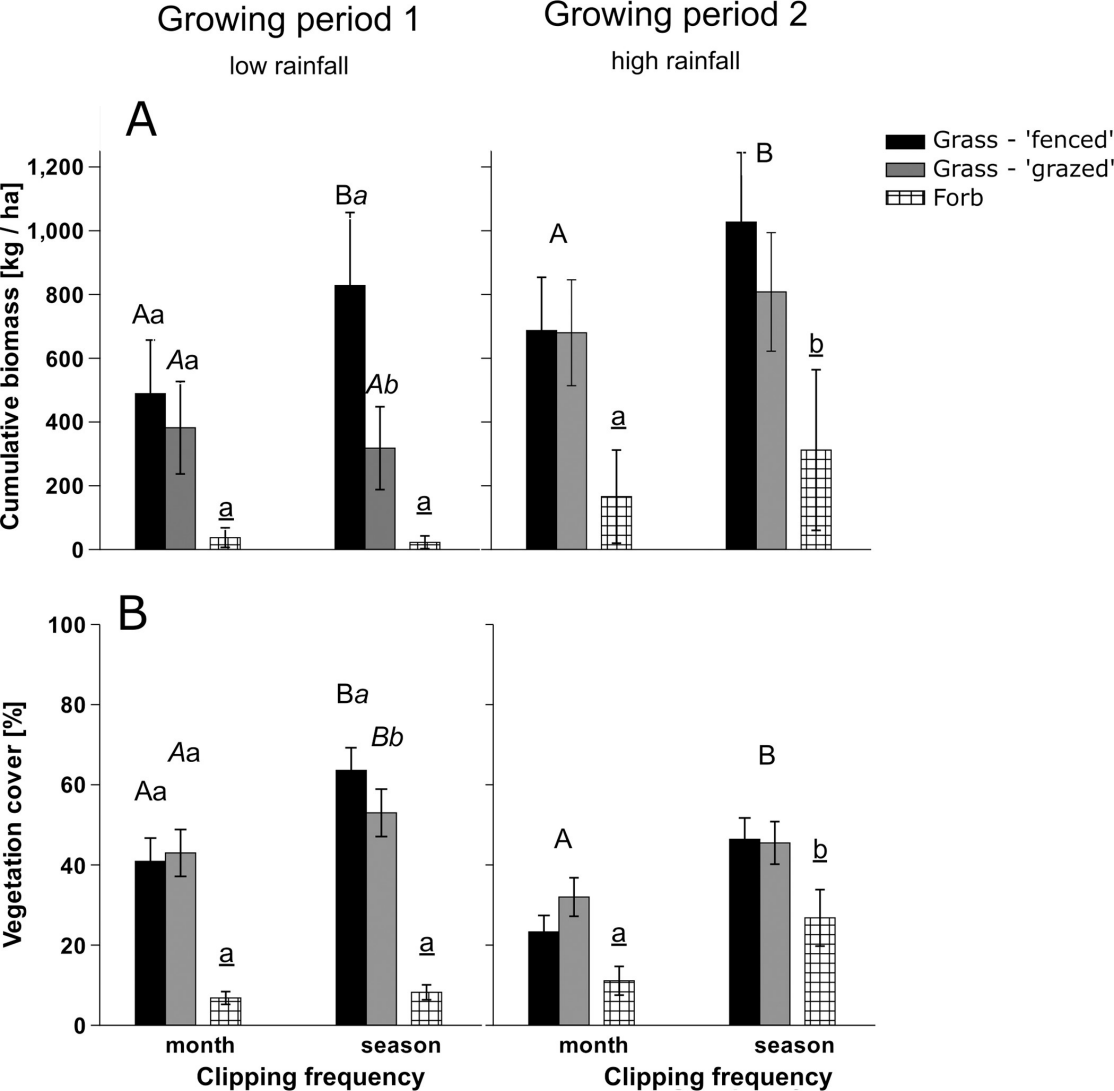
Cumulative biomass and vegetation cover in experimental plots. **(A)**–Cumulative mean grass and forb biomass [kg/ha] and **(B)** mean grass and forb cover at the end of the growing period [%]) in experimental plots that were clipped each month (‘month’) or once after the growing period (‘season’), in the rangelands of the community of Loibor Siret, Tanzania. Half of the quadrats, containing the plots, were fenced to exclude herbivores (‘fenced’), the other half were open for grazing (‘grazed’). The graphs show the results of two growing periods: Growing period 1 (March- June 2019) and Growing period 2 (November 2019 –January 2020). Growing period 1: Significant interaction between clipping frequency and herbivory for grass biomass and grass cover. Significant differences (p<0.05) are indicated by different letters of same style. Capital letters indicate treatment effects of clipping frequency on grass biomass. Small letters indicate differences of grass biomass between fenced and grazed plots within the clipping frequency. Underlined, small letters refer to differences in forb biomass. Vertical bars represent ± 1 s.e..

Excluding herbivores affected the vegetation parameter significantly during growing period 1, when rainfall was low, but then only in seasonally clipped plots. Here the proportion of bare ground was between 28 and 52% lower in fenced plots compared to grazed plots (Tukey’s post- hoc, *p* < 0.002, Fig 2 and Table 1). Grass regrowth was positive in fenced, seasonally clipped plots, while the other treatments showed predominantly negative and significantly lower grass regrowth (Tukey’s post- hoc, *p* = 0.001; Fig 3A). Grass biomass was 2.6 times higher and grass cover 20% higher in fenced, seasonally clipped plots compared to grazed, seasonally clipped plots (Tukey’s post- hoc, *p* < 0.001 and *p* = 0.017, respectively, Fig 4 and Table 2). Monthly clipped plots had low regrowth and vegetation cover throughout and showed overall high proportion of bare ground that did not differ among fenced and grazed plots (Figs 2–4). During growing period 2, when rainfall was high, mean grass regrowth was seven times higher when herbivores were excluded (F_1,26_ = 5.04, *p* = 0.03; Fig 3 and Table 1), but herbivory had little effect on the remaining parameter. Forb regrowth was mostly negative during low rainfall in growing period 1 but positive in all seasonally clipped plots during high rainfall in growing period 2.

### Higher clipping frequency reduces grass cover, regrowth and biomass (H3)

Low clipping frequency applied to seasonally clipped plots resulted in lower proportion of bare ground, higher regrowth rates and higher vegetation biomass and cover compared to monthly clipped plots (Figs 2–4).

In growing period 1, the proportion of bare ground was between 7% and 77% lower and grass cover 23% and 57% higher in seasonally clipped plots compared to monthly clipped plots (Tukey’s post- hoc, proportion bare ground grazed plots in DryRL *p* = 0.08, grazed plots in RainRL *p* = 0.34 and all other combinations *p* < 0.001; grass cover fenced *p* < 0.001 and grazed *p* = 0.02; Figs 2 and 4). Fenced, seasonally clipped plots showed 69% higher grass biomass (Tukey’s post- hoc, *p* = 0.002, Fig 4) compared to fenced, monthly clipped plots. Grass regrowth of fenced, seasonally clipped plots was significantly higher and positive compared to fenced, monthly clipped plots (Post- hoc, *p* < 0.001, Fig 4A, see results above).

In growing period 2, the proportion of bare ground was between 37% and 85% lower in seasonally clipped plots vs. monthly clipped plots (Post- hoc, *p<* 0.05 for all combinations, Fig 1). Grass and forb biomass in seasonally clipped plots were 34% and 88% higher compared to monthly clipped plots (F_1,26_ = 8.61, *p* = 0.007 and F_1,30_ = 6.37 *p =* 0.02, respectively, Fig 4A). Grass cover was 1.6 times higher and forb cover 2.4 times higher in seasonally clipped plots compared to monthly clipped plots across herbivory and rangeland area (Chisq = 24.92, *p* < 0.001 and Chisq = 33.70, *p* < 0.001, respectively, Fig 4B and Table 1). Grass and forb regrowth were significantly higher in seasonally clipped plots compared to monthly clipped plots (F_1,26_ = 8.34 *p =* 0.008 and F_1,26_ = 12.24 *p =* 0.002, respectively, Fig 2A). Grass was the dominant vegetation form in our plots across all treatments and throughout both growing periods.

## Discussion

### Rainy season rangeland shows lowest plant cover and regrowth capacity (H1)

As hypothesized, the resilience of the herbaceous vegetation towards intense disturbance, applied as clipping vegetation, was the lowest in the RainRL compared to the DryRL and Alalili. The vegetation of the RainRL could not compensate for the biomass removal, not even during the wetter growing period or at a low clipping frequency. Rainy season rangeland is grazed during the rainy season and rested during the dry season, when there is no or little water available for vegetation growth after defoliation by livestock [30]. Even though our study area is used by wildlife as rainy season grazing land and a co-evolution of grasses and herbivores is thus very likely, increased grazing pressure due to livestock usage might have exceeded the grazing tolerance and resistance of the grass community [61, 62]. Our results support other studies that find recovery during dry periods is less effective in restoring ecosystem services of rangelands, e.g. grass biomass [13]. From our results, RainRL is the most sensitive rangeland grazing practice to climatic variation and increasing disturbance intensity with low recovery potential. Recommendations on sustainable management of semi- arid rangelands highlight the effectiveness of rainy season resting, thus dry season rangeland, as a recovery strategy [12, 13]. We concur with this recommendation, as in our study DryRL and Alalili exhibited the lowest proportion of bare ground and the highest grass regrowth capacity, irrespective of available rainfall or clipping frequency. The concept of seasonal exclosures, such as Alalili, is widespread in pastoral communities of eastern Africa, due to its positive effect on vegetation cover, grass biomass and biodiversity [8, 14, 16, 17]. Our study confirms not only that seasonal resting is beneficial but also that rangeland rested during the rainy season inherits higher resilience towards intense disturbance, even if the latter occurs at a higher frequency. This is of particular interest since rising livestock numbers and extreme weather events will most likely increase the pressure on the rangeland in the future [35, 63]. In contrast to the DryRL, we emphasize that the RainRL is the component that is limiting the resilience of the rangeland system. Management concepts for RainRL must therefore imply sufficient recovery time after heavy defoliation, from our data, at least one growing period. Particularly during low rainfall years, grazing pressure on RainRL should be restricted to a minimum, e.g. by increasing RainRL area or decreasing livestock numbers. Our findings highlight the importance of spatio-temporal movement in semi-arid rangelands allowing recovery time after livestock disturbance [3, 10, 36] and suggest that the concept is applicable on a small scale, if specific requirements of each rangeland area can be incorporated in the grazing management.

### Herbivory affects vegetation parameters strongly when rainfall is low (H2)

During growing period 1, when overall rainfall was low, the herbaceous vegetation of our experimental plots could not maintain high biomass production or ground cover under herbivory, and recovery to the original biomass was hardly possible. Herbivory compared to herbivore exclusion can enhance plant regrowth under moderate defoliation and low clipping frequency [23]. However, herbivore exclusion is an established and approved tool to restore heavily used and even overused rangelands [29]. In our study, only low clipping frequency and complete exclusion of herbivory seemed to be an appropriate treatment when rainfall was limiting. Negative effects of herbivory on the rangeland condition were less visible during growing period 2, when rainfall was exceptionally high. These results align with [47], who propose that rainfall is a key factor influencing compensatory growth after defoliation, often overriding effects of herbivory [20]. Rangelands receiving higher rainfall show higher productivity and higher recovery potential [20, 27, 64]. During dry conditions, by contrast, long-lasting overgrazing impacts may occur through even a short grazing duration at high stocking rates [65]. Coping strategies to deal with limited rainfall can include livestock stocking rates that are adapted to current rainfall conditions and seasonal forage supply, hence to make use of good rainfall years and avoid overgrazing during dry conditions [12]. The Alalili of our study show lowest proportion of bare ground during low rainfall during growing period 1, which confirms that the concept of seasonal exclosures can be another successful management component to cope with drought conditions when grazing resources are depleted otherwise and highlights the importance to conserve seasonal exclosures [8].

### Higher clipping frequency reduces grass biomass, regrowth and cover (H3)

As expected, severe and frequent defoliation, as simulated in our monthly clipping plots, negatively influenced the recovery potential of the herbaceous vegetation and reduced plant biomass and vegetation cover, which are crucial in maintaining high water infiltration rates and soil moisture and combating soil erosion [66, 67]. Reduced vegetation cover caused by defoliation through clipping is in accordance with other studies [43, 68]. Vegetation cover helps to buffer erratic and heavy rainfall events and can represent a measure of resilience in a disturbed system [20, 63]. Critical vegetation cover to protect the rangeland from erosion can vary in semi-arid rangelands, yet, vegetation cover decreasing below 40% ground cover was proposed as one indicator for soil degradation in rangelands experiencing temporal and spatial rainfall variability [27, 69] while vegetation cover higher than 50–75% was referred to as sustainable to maintain soil functions [70]. The vegetation cover of our seasonally clipped plots was considerably higher than in our monthly clipped plots and rarely fell below 60%, despite high intensity of biomass removal. Once ground cover is high, vegetation is likely not as strongly affected by further disturbance in the future [20].

Rapid vegetation regrowth after defoliation as a compensation mechanism can lead to even increased forage provision [28]. Our results confirm, however, that vegetation regrowth strongly depends on the timing and intensity of defoliation as well as the recovery time [28, 43]. While increasing clipping frequency often leads to decreasing biomass, sufficient recovery time between clipping events seem to alleviate the impact and lead to higher yield for grazers [18, 19, 28]. Our results suggest that recovery time after heavy disturbance should generally not be less than one growing period, to guarantee continuous forage provision and to stay within the capacity of the rangeland to recover from disturbances.

In our study, forb biomass increased visibly during the second growing period. In semi-arid rangelands forbs usually cope better with high grazing pressure [16]. Additionally, they have a higher potential to endure drought conditions and tend to outcompete grasses under higher rainfall variability [71, 72]. It seems likely that forbs benefitted from the combination of low rainfall and intense disturbance, followed by a rain intense growing period during our study. Although forbs can be valuable components of herbaceous communities in rangelands and for medicinal or ceremonial purposes, grasses and sedges are usually preferred by livestock [73–75]. Higher abundance of forbs has been interpreted as an indicator of low rangeland conditions [76]. Invasive forbs often establish following overutilization of rangelands, leaving them unsuitable for livestock grazing [9]. While we did not investigate the spread of invasive plant species, our study region had invasives such as *Solanum campylacanthum* [45]. We recommend intense monitoring of forb species dynamics, particularly invasive plants in our study region as we claim that this region might become more susceptible to forb dominance, as livestock pressure and rainfall variability are predicted to increase [35, 63]. The results of our study show the risk of frequent grazing events during times of drought, which will not only increase bare soil percentage and decrease grass productivity but might eventually shift the herbaceous community towards less desired forb- dominated compositions [76].

## Conclusion

We conclude that traditionally implemented seasonal migration, is essential for sustaining rangeland productivity and resilience under increasing intensity of disturbance. Resting time after intense defoliation should be a key management strategy to maintain this capacity of the rangelands. We highlight that rangeland grazed during the rainy season need higher management attention, but we found that the current traditional grazing management was capable of sustaining herbaceous forage production in our study site, in particular when rainfall was not limiting. Highly erratic rainfall events will, however, challenge these rangelands in the future. Thus, we propose that livestock grazing pressure and resting time after intense defoliation must be adapted to rainfall related rangeland conditions, e.g. by decreasing livestock numbers and extending the area of RainRL, particularly when rainfall is low. Seasonal exclosures, such as Alalili, as a strategy to cope with drought situations should be conserved and be part of the overall management plan. To capture the intense and changing rainfall pattern and its impact on the vegetation, we suggest long-term monitoring of the vegetation, its composition and the development of the bare ground proportion. Follow-up studies on the rangelands’ recovery potential under actual current livestock grazing rather than using clipping only might help to gain deeper understanding of increasing disturbance through herbivory. The results of our study are likely applicable to other semi-arid rangelands that are traditionally managed, i.e., use seasonal resting of vegetation and enclosures. Confounding factors might include changes of land ownership, land use and political decisions and need close monitoring in the future.

## Supporting information

S1 Table Information on the full statistical models, including fixed and random effects. (PDF)

S1 Data Growing period 1_2_biomass, bare ground, regrowth. (XLSX)
