# Supplementary material for: Love your wet grass! Dry season grazing reserves show highest grass regrowth in communal semi-arid rangelands of Tanzania
Source: PLoS One. 2024 Nov 18;19(11):e0313818. doi: 10.1371/journal.pone.0313818 (PMC11573159; doi:10.1371/journal.pone.0313818)
Supplement: S1 Table — (PDF) [file pone.0313818.s001.pdf]

Love your wet grass! Dry season grazing reserves show highest grass regrowth in communal semi-arid rangelands of Tanzania

Sabine A. Baumgartner[1], Stuart W. Smith[2,3], Gundula S. Bartzke[4], Oloshiro Laizar[5], Jane F. Ploechl[6], Lena M. Michler[1], Elizabeth Naro[7], Anna C. Treydte[1,2]

1 Department of Ecology of Tropical Agricultural Systems (490f), Institute of Agricultural Sciences in the Tropics (Hans-Ruthenberg-Institute), University of Hohenheim, 70599 Stuttgart, Germany

2 Department of Physical Geography, Stockholm University, 106 91 Stockholm, Sweden.

3 Ecological Sciences, The James Hutton Institute, Craigiebuckler, Aberdeen, AB15 8QH, UK

4 Biostatistics Unit, Institute of Crop Science, University of Hohenheim, 70599 Stuttgart, Germany

5 Grazing Committee, Village of Loibor Siret, Tanzania

6 Istituto Oikos, Haile Selassie Road n. 12, House Plot 165, Arusha, P.O. Box 8342, Tanzania

7 African People & Wildlife Fund, PO Box 624, Bernardsville, NJ 07924, USA

Information on full statistical models including fixed and random effects

Response variable	GP	AIC	Fixed effects	Estimate	Std. error	Random effects	Variance	Std. dev.
Grass biomass	GP1	1265	Intercept	36.10	8.28	Quadrat(block:site)	28.15	5.31
			RangelandArea DryRL	-10.80	11.65	Block:site	15.05	3.88
			RangelandArea RainRL	-8.34	11.65	Site	115.26	10.74
			herbivory	-1.88	3.40	Residual	25.76	5.08
			clippingFrequency	7.55	3.40			
			RangelandArea DryRL:herbivory	-3.01	4.16			
			RangelandArea RainRL:herbivory	-1.30	4.16			
			RangelandArea DryRL:clippingFrequency	4.49	4.16			
			RangelandArea RainRL:clippingFrequency	-2.38	4.16			
			herbivory:clippingFrequency	-10.50	3.40			
	GP2	1005	Intercept	23.92	3.92	Quadrat(block:site)	7.79	2.79
			RangelandArea DryRL	3.67	5.50	Block:site	0.77	0.88
			RangelandArea RainRL	-3.04	5.58	Site	25.94	5.09
			herbivory	-2.77	1.90	Residual	11.28	3.36
			clippingFrequency	4.47	1.90			
			RangelandArea DryRL:herbivory	3.79	2.30			
			RangelandArea RainRL:herbivory	4.22	2.49			
			RangelandArea DryRL:clippingFrequency	0.88	2.30			
			RangelandArea RainRL:clippingFrequency	-1.93	2.49			
			herbivory:clippingFrequency	-2.42	1.96			

Response variable	GP	AIC	Fixed effects	Estimate	Std. error	Random effects	Variance	Std. dev.
Forb biomass	GP1	725	Intercept	4.02	1.30	Quadrat(block:site)	0.87	0.93
			RangelandArea DryRL	-1.18	1.82	Block:site	0.39	0.62
			RangelandArea RainRL	-1.16	1.82	Site	2.65	1.63
			herbivory	-0.33	0.65	Residual	1.29	
			clippingFrequency	-0.08	0.65			
			RangelandArea DryRL:herbivory	0.61	0.80			
			RangelandArea RainRL:herbivory	-0.33	0.80			
			RangelandArea DryRL:clippingFrequency	0.57	0.80			
			RangelandArea RainRL:clippingFrequency	0.06	0.80			
			herbivory:clippingFrequency	-0.56	0.65			
	GP2	943	Intercept	13.51	4.35	Quadrat(block:site)	3.28	1.81
			RangelandArea DryRL	-5.84	6.13	Block:site	0.24	0.49
			RangelandArea RainRL	-4.52	6.17	Site	35.37	5.95
			herbivory	-3.10	1.39	Residual	3.06	
			clippingFrequency	2.76	1.39			
			RangelandArea DryRL:herbivory	2.72	1.68			
			RangelandArea RainRL:herbivory	3.29	1.81			
			RangelandArea DryRL:clippingFrequency	2.54	1.68			
			RangelandArea RainRL:clippingFrequency	-1.84	1.81			
			herbivory:clippingFrequency	0.03	1.43			

Response variable	GP	AIC	Fixed effects			Random effects		
				Estimate	*Std. error*		*Variance*	*Std. dev.*
Grass cover	GP1	-252	Intercept	0.14	0.47	Quadrat(block:site)	0.17	0.42
			RangelandArea DryRL	-0.44	0.66	Block:site	0.16	0.40
			RangelandArea RainRL	-1.28	0.66	Site	0.27	0.52
			herbivory	0.10	0.28			
			clippingFrequency	1.41	0.28			
			RangelandArea DryRL:herbivory	0.04	0.34			
			RangelandArea RainRL:herbivory	-0.04	0.34			
			RangelandArea DryRL:clippingFrequency	-0.57	0.34			
			RangelandArea RainRL:clippingFrequency	-0.41	0.34			
			herbivory:clippingFrequency	-0.61	0.28			
	GP2	-135	Intercept	-1.47	0.39	Quadrat(block:site)	0.24	0.49
			RangelandArea DryRL	0.56	0.53	Block:site	0.03	0.17
			RangelandArea RainRL	-0.06	0.56	Site	0.13	0.36
			herbivory	0.56	0.35			
			clippingFrequency	1.13	0.35			
			RangelandArea DryRL:herbivory	-0.06	0.41			
			RangelandArea RainRL:herbivory	-0.20	0.45			
			RangelandArea DryRL:clippingFrequency	0.10	0.41			
			RangelandArea RainRL:clippingFrequency	-0.09	0.45			
			herbivory:clippingFrequency	-0.51	0.35			

Response variable	GP	AIC	Fixed effects			Random effects		
				Estimate	*Std. error*		*Variance*	*Std. dev.*
Forb cover	GP1	-670	Intercept	-2.63	0.46	Quadrat(block:site)	0.15	0.38
			RangelandArea DryRL	-0.18	0.65	Block:site	0.10	0.32
			RangelandArea RainRL	-0.40	0.65	Site	0.26	0.51
			herbivory	0.03	0.30			
			clippingFrequency	0.39	0.29			
			RangelandArea DryRL:herbivory	-0.01	0.36			
			RangelandArea RainRL:herbivory	-0.10	0.37			
			RangelandArea DryRL:clippingFrequency	0.32	0.36			
			RangelandArea RainRL:clippingFrequency	-0.31	0.37			
			herbivory:clippingFrequency	-0.36	0.30			
	GP2	-355	Intercept	-1.01	0.64	Quadrat(block:site)	0.24	0.49
			RangelandArea DryRL	-1.33	0.90	Block:site	0.00	0.00
			RangelandArea RainRL	-1.51	0.92	Site	0.66	0.81
			herbivory	-0.88	0.36			
			clippingFrequency	1.11	0.36			
			RangelandArea DryRL:herbivory	0.79	0.44			
			RangelandArea RainRL:herbivory	1.07	0.49			
			RangelandArea DryRL:clippingFrequency	0.28	0.44			
			RangelandArea RainRL:clippingFrequency	-0.33	0.49			
			herbivory:clippingFrequency	-0.03	0.38			

Response variable	GP	AIC	Fixed effects			Random effects		
				Estimate	*Std. error*		*Variance*	*Std. dev.*
Proportion Bare ground	GP1	-293	Intercept	-0.45	0.38	Quadrat(block:site)	0.12	0.34
			RangelandArea DryRL	0.36	0.53	Block:site	0.09	0.29
			RangelandArea RainRL	1.34	0.53	Site	0.18	0.42
			herbivory	-0.01	0.24			
			clippingFrequency	-2.01	0.25			
			RangelandArea DryRL:herbivory	-0.06	0.30			
			RangelandArea RainRL:herbivory	-0.13	0.30			
			RangelandArea DryRL:clippingFrequency	0.68	0.30			
			RangelandArea RainRL:clippingFrequency	0.87	0.30			
			herbivory:clippingFrequency	0.91	0.24			
	GP2	-248	Intercept	0.12	0.24	Quadrat(block:site)	0.18	0.43
			RangelandArea DryRL	0.55	0.33	Block:site	0.001	0.03
			RangelandArea RainRL	1.15	0.35	Site	0.01	0.08
			herbivory	0.27	0.30			
			clippingFrequency	-2.65	0.32			
			RangelandArea DryRL:herbivory	-0.79	0.38			
			RangelandArea RainRL:herbivory	-0.96	0.40			
			RangelandArea DryRL:clippingFrequency	-0.28	0.38			
			RangelandArea RainRL:clippingFrequency	0.73	0.40			
			herbivory:clippingFrequency	0.91	0.32			

Response variable	GP	AIC	Fixed effects			Random effects		
				Estimate	*Std. error*		*Variance*	*Std. dev.*
Grass regrowth	GP1	2694	Intercept	58.79	251.52	Quadrat(block:site)	82306.00	286.90
			RangelandArea DryRL	-360.41	349.70	Block:site	0.00	0.00
			RangelandArea RainRL	-358.04	349.70	Site	84172.00	290.10
			herbivory	-167.94	184.07	Residual	77341.00	278.10
			clippingFrequency	363.16	184.07			
			RangelandArea DryRL:herbivory	27.84	225.43			
			RangelandArea RainRL:herbivory	44.51	225.43			
			RangelandArea DryRL:clippingFrequency	234.76	225.43			
			RangelandArea RainRL:clippingFrequency	71.29	225.43			
			herbivory:clippingFrequency	-511.29	184.07			
	GP2	2533	Intercept	74.63	171.67	Quadrat(block:site)	70570.00	265.60
			RangelandArea DryRL	325.50	232.85	Block:site	30500.00	174.60
			RangelandArea RainRL	-397.12	251.51	Site	0.00	0.00
			herbivory	-301.25	188.23	Residual	133400.00	365.30
			clippingFrequency	359.17	188.23			
			RangelandArea DryRL:herbivory	316.13	227.95			
			RangelandArea RainRL:herbivory	296.43	246.22			
			RangelandArea DryRL:clippingFrequency	221.12	227.95			
			RangelandArea RainRL:clippingFrequency	-78.75	246.22			
			herbivory:clippingFrequency	-246.51	194.40			

Response variable	GP	AIC	Fixed effects	Estimate	Std. error	Random effects	Variance	Std. dev.
Forb regrowth	GP1	2494	Intercept	-357.71	77.85	Quadrat(block:site)	4049.00	63.63
			RangelandArea DryRL	248.16	107.78	Block:site	9843.00	99.21
			RangelandArea RainRL	210.72	107.78	Site	2167.00	46.55
			herbivory	140.23	63.45	Residual	32111.00	179.20
			clippingFrequency	81.76	63.45			
			RangelandArea DryRL:herbivory	-184.44	77.71			
			RangelandArea RainRL:herbivory	-104.21	77.71			
			RangelandArea DryRL:clippingFrequency	56.18	77.71			
			RangelandArea RainRL:clippingFrequency	-70.07	77.71			
			herbivory:clippingFrequency	-81.82	63.45			
	GP2	2439	Intercept	-18.17	138.96	Quadrat(block:site)	22412.20	149.71
			RangelandArea DryRL	-45.74	191.46	Block:site	928.30	30.47
			RangelandArea RainRL	60.16	199.43	Site	20004.50	141.44
			herbivory	-37.03	121.31	Residual	83016.90	288.13
			clippingFrequency	300.75	121.31			
			RangelandArea DryRL:herbivory	-0.08	146.91			
			RangelandArea RainRL:herbivory	163.06	158.68			
			RangelandArea DryRL:clippingFrequency	154.93	146.91			
			RangelandArea RainRL:clippingFrequency	-257.75	158.68			
			herbivory:clippingFrequency	-90.63	125.29			
